# Human phenotype ontology annotation and cluster analysis to unravel genetic defects in 707 cases with unexplained bleeding and platelet disorders

**DOI:** 10.1186/s13073-015-0151-5

**Published:** 2015-04-09

**Authors:** Sarah K Westbury, Ernest Turro, Daniel Greene, Claire Lentaigne, Anne M Kelly, Tadbir K Bariana, Ilenia Simeoni, Xavier Pillois, Antony Attwood, Steve Austin, Sjoert BG Jansen, Tamam Bakchoul, Abi Crisp-Hihn, Wendy N Erber, Rémi Favier, Nicola Foad, Michael Gattens, Jennifer D Jolley, Ri Liesner, Stuart Meacham, Carolyn M Millar, Alan T Nurden, Kathelijne Peerlinck, David J Perry, Pawan Poudel, Sol Schulman, Harald Schulze, Jonathan C Stephens, Bruce Furie, Peter N Robinson, Chris van Geet, Augusto Rendon, Keith Gomez, Michael A Laffan, Michele P Lambert, Paquita Nurden, Willem H Ouwehand, Sylvia Richardson, Andrew D Mumford, Kathleen Freson, on behalf of the BRIDGE-BPD Consortium

**Affiliations:** School of Clinical Sciences, University of Bristol, Bristol, UK; Department of Haematology, University of Cambridge, Cambridge Biomedical Campus, Cambridge, UK; NHS Blood and Transplant, Cambridge Biomedical Campus, Cambridge, UK; Centre for Haematology, Hammersmith Campus, Imperial College Academic Health Sciences Centre, Imperial College London, London, UK; Imperial College Healthcare NHS Trust, DuCane Road, London, UK; Department of Haematology, University College London Cancer Institute, London, UK; The Katharine Dormandy Haemophilia Centre and Thrombosis Unit, Royal Free London NHS Foundation Trust, London, UK; Medical Research Council Biostatistics Unit, Cambridge Biomedical Campus, Cambridge, UK; Institut Hospitalo-Universitaire LIRYC, PTIB, Hôpital Xavier Arnozan, Pessac, France; Department of Haematology, Guys and St Thomas’ NHS Foundation Trust, London, UK; Institut für Immunologie und Transfusionsmedizin Universitätsmedizin Ernst-Moritz-Arndt Universität, Greifswald, Germany; Pathology and Laboratory Medicine, University of Western Australia, Crawley, WA Australia; Haematological Laboratory, Trousseau Children’s Hospital and INsermU1009, Paris, France; Department of Haematology, Addenbrooke’s Hospital, Cambridge University Hospitals NHS Foundation Trust, Cambridge Biomedical Campus, Cambridge, UK; Department of Haematology, Great Ormond Street Hospital for Children NHS Trust, London, UK; Center for Molecular and Vascular Biology, University of Leuven, Leuven, Belgium; Beth Israel Deaconess Medical Centre, Harvard Medical School, Boston, USA; Lehrstuhl für Experimentelle Biomedizin, Universitätsklinikum Würzburg, Würzburg, Germany; Institut für Medizinische Genetik und Humangenetik, Charité Universitätsmedizin, Berlin, Germany; Max Planck Institute for Molecular Genetics, Berlin, Germany; Institute for Bioinformatics, Department of Mathematics and Computer Science Freie Universität, Berlin, Germany; Division of Hematology, Children’s Hospital of Philadelphia, Philadelphia, USA; Department of Pediatrics, Perelman School of Medicine at the University of Pennsylvania, Philadelphia, USA; Wellcome Trust Sanger Institute, Wellcome Trust Genome Campus, Hinxton, Cambridge, UK; School of Cellular and Molecular Medicine, University of Bristol, Bristol, UK

## Abstract

**Background:**

Heritable bleeding and platelet disorders (BPD) are heterogeneous and frequently have an unknown genetic basis. The BRIDGE-BPD study aims to discover new causal genes for BPD by high throughput sequencing using cluster analyses based on improved and standardised deep, multi-system phenotyping of cases.

**Methods:**

We report a new approach in which the clinical and laboratory characteristics of BPD cases are annotated with adapted Human Phenotype Ontology (HPO) terms. Cluster analyses are then used to characterise groups of cases with similar HPO terms and variants in the same genes.

**Results:**

We show that 60% of index cases with heritable BPD enrolled at 10 European or US centres were annotated with HPO terms indicating abnormalities in organ systems other than blood or blood-forming tissues, particularly the nervous system. Cases within pedigrees clustered closely together on the bases of their HPO-coded phenotypes, as did cases sharing several clinically suspected syndromic disorders. Cases subsequently found to harbour variants in *ACTN1* also clustered closely, even though diagnosis of this recently described disorder was not possible using only the clinical and laboratory data available to the enrolling clinician.

**Conclusions:**

These findings validate our novel HPO-based phenotype clustering methodology for known BPD, thus providing a new discovery tool for BPD of unknown genetic basis. This approach will also be relevant for other rare diseases with significant genetic heterogeneity.

**Electronic supplementary material:**

The online version of this article (doi:10.1186/s13073-015-0151-5) contains supplementary material, which is available to authorized users.

## Background

Bleeding and platelet disorders (BPD) are a heterogeneous group of rare diseases caused by abnormalities of coagulation factors, platelets or blood vessel walls. Although in most cases the major clinical feature is abnormal bleeding, BPD are frequently associated with phenotypes in other organ systems, particularly the immune system (for example, Wiskott-Aldrich syndrome (WAS, ORPHA906)), skeleton (for example, Thrombocytopenia with Absent Radius syndrome (ORPHA3320)), eye (for example, Hermansky-Pudlak Syndrome (HPS, ORPHA79430)) and kidney (for example, *MYH9*-related disorder (MYH9-RD, ORPHA182050)). BPD may be inherited as autosomal recessive, autosomal dominant, X-linked, or complex traits. Collectively, BPD represent a significant diagnostic and management challenge to health care systems [1].

Diagnosis of BPD currently requires clinical evaluation, then tests such as coagulation factor activity and platelet function assays [2,3]. However, for mild BPD, this enables diagnosis to the level of a defective coagulation factor or a platelet pathway in only 40% to 60% of cases [4,5]. The proportion of cases with BPD that receive genetic diagnosis is even lower [6], partly because the genetic basis of most BPD remains unknown, particularly for prevalent sub-groups such as platelet secretion defects [5]. Even for BPD with a known genetic basis, diagnosis may not be possible if phenotype tests are insufficiently specific to point towards relevant genes. Moreover, many BPD with causal variants in the same gene are clinically heterogeneous, as illustrated by MYH9-RD in which macrothrombocytopenia is a consistent feature, but neutrophil Döhle-like inclusions, renal impairment, deafness and cataracts are variable [7,8]. High throughput sequencing (HTS) has the potential to circumvent these limitations by providing a diagnostic tool for genetic diagnosis and by enabling gene discovery to increase the repertoire of disorders for which genetic diagnosis is possible.

Analysis of large case collections recruited through consortia of investigators and technologies such as HTS assist discovery of rare BPD genes [9,10]. However, gene discovery additionally requires analysis of shared data through systematic phenotype coding to identify similarities between unrelated cases. Phenotype coding also facilitates creation of clinical registries, genotype-phenotype databases and biobanks. Examples of coding systems include the World Health Organization International Classification of Diseases (ICD) [11], which provides a post-diagnosis classification of disease organized by organ system that is unsuitable for coding rare genetic diseases particularly those that affect multiple organs. The International Health Terminology Standards Development Organisation Systematized Nomenclature of Medicine Clinical Terminology (SNOMED-CT) [12] is an alternative system, but is not optimised for laboratory results and has greater emphasis on specific diseases rather than phenotypes. Platforms such as Online Mendelian Inheritance in Man (OMIM) [13] and Orphanet [14] provide descriptions of known genetic disorders but do not utilise systematic phenotype terms. The Bleeding History Phenotype Ontology [15] is a hierarchical ontology system that may enable phenotypic similarities between some cases with BPD to be resolved. However, this ontology has a limited repertoire of phenotypic terms outside those immediately pertinent to bleeding. Moreover, there are no terms for the results of laboratory tests such as light transmission aggregation or dense granule secretion. These are essential for the accurate phenotypic description of platelet function disorders, which are the largest single group of BPD of unknown genetic basis.

The Human Phenotype Ontology (HPO) project is an international initiative to support the phenotypic annotation of genetic disorders available under an open-source licence [16]. The HPO version 887 contains a set of 10,371 terms that describe abnormalities of human phenotype and 13,556 relations between the HPO terms organised hierarchically through *is-a* relations [17]. For example, ‘thrombocytopenia is-a abnormal platelet count is-a abnormality of thrombocytes’. This enables phenotypes to be described using a standardised and controlled vocabulary with greater detail and flexibility than other coding systems. The HPO has previously been applied to diseases using phenotypic terms from Orphanet and OMIM [18] but it has not previously been used to describe and compare phenotype data from individual cases. Systematic comparison of phenotype data from individual cases assists gene discovery because clusters of cases with similar phenotypes are likely to share defects in the same gene or interacting set of genes [19].

The BRIDGE-BPD study [20] is a multicentre, observational consortium study that aims to identify causal gene defects in cases with BPD of unknown aetiology by deep multi-system phenotyping and HTS. We report the results of Stage 1 of the BRIDGE-BPD study in which we develop new HPO terminology to facilitate annotation of BPD phenotypes in 707 BPD cases recruited at 10 European and US centres. We show for the first time how HPO annotation can be used to describe the phenotypes of individual cases within a large rare disease collection and how a novel statistical clustering approach using HPO data guides gene discovery.

## Methods

### Study overview and enrolment criteria

The target population for the BRIDGE-BPD study comprises children and adults with disorders of platelet number, volume, morphology or function or with pathological bleeding that cannot be explained by standard laboratory tests. To ensure enrolment of cases with a high likelihood of a genetic BPD, the inclusion criteria require features such as a BPD from an early age, a BPD that is part of a syndromic disorder or a family history of a BPD. Cases with an acquired BPD (Table 1) or a known genetic BPD are excluded unless they display bleeding or laboratory features that cannot be explained by this diagnosis alone. For example, a case with haemophilia A and unexplained thrombocytopenia is considered eligible on the basis of thrombocytopenia. Most cases had already been investigated within research and specialist clinical diagnostic facilities, maximising exclusion of acquired or known genetic BPD. The BRIDGE-BPD study is approved by a UK Research Ethics Committee (Cambridgeshire 1 Research Ethics Committee 10/H0304/66) and appropriate national ethics authorities for non-UK enrolment centres (Additional file 1). All study procedures were performed after the participants provided informed written consent and were in accordance with the Declaration of Helsinki. Sequence data for cases who provided consent for public access have been deposited at the European Genome-Phenome Archive (EGAS00001001172).

**Table 1:** **Eligibility criteria for BRIDGE bleeding and platelet disorders study**

**Inclusion criteria**	**Exclusion criteria**
Platelet count less than 100 × 10^9^/L or greater than 400 × 10^9^/L, or	Acquired bleeding or platelet disorders, including any of the following:
Mean platelet volume less than 6 fL or greater than 12 fL, or	Use of any medication known to affect platelet function or cause bleeding
Reproducible abnormal platelet function test results, or	Immune thrombocytopenia
Abnormal platelet morphology by light or electron microscopy, or	HIV infection
Pathological bleeding of unknown aetiology, and	Malignancies, particularly those affecting haemopoiesis
Considered by referring clinician to be of genetic aetiology	Bone marrow aplasia
	Thrombotic thrombocytopenic purpura/ Haemolytic-uremic syndrome
	Acute viral infection
	Splenomegaly
	Uraemia or hepatic failure

### Recruitment of cases and collection of phenotype data

Index cases and pedigree members were identified by screening clinic lists, case notes and local registries. Potential participants were checked for eligibility and were invited to give informed written consent. Demographic and clinical data, laboratory test results and pedigree relationships were recorded on a case report form.

### BRIDGE-BPD database

Pseudonymised demographic and clinical data were transferred to electronic data capture pages usually as quantitative or categorical variables but with a minority as free text to maintain detail. Bleeding symptoms were recorded as numerical severity scores for the 12 major symptoms within the MCMDM-1 VWD Bleeding Assessment Tool [21], or as the terms ‘yes’ or ‘no’ for each symptom (Additional file 2). Laboratory test results were recorded as quantitative variables, except for tests such as platelet light transmission aggregation (LTA) and ATP secretion, which were recorded as ‘normal’ or ‘abnormal’ according to the interpretation of the enrolling clinician. Platelet morphology determined by light or electron microscopy was recorded as free text.

### Development of HPO terminology for BPD

In order to ensure accurate annotation of the bleeding and platelet phenotype, 80 terms and associated *is-a* relationships were added to HPO [17], in parallel with development of hpoPlot, a new free software tool that summarises HPO codes of a set of cases, released on the Comprehensive R Archive Network [22]. The HPO modifications were predominantly within the abnormality of blood and blood-forming tissue leading class and its constituent classes ‘abnormality of thrombocytes’, ‘abnormal bleeding’, ‘abnormality of coagulation’ and ‘abnormal thrombosis’. Some terms required creation of a new ‘abnormal platelet morphology’ class (Additional file 3). Many terms within ‘abnormality of blood and blood-forming tissue’ overlap with other system-specific HPO leading classes, particularly ‘abnormality of the immune system’ (Additional file 4).

### Automated and suggested HPO terms

To improve the reliability and efficiency of HPO coding, cases were annotated with relevant terms automatically for abnormal bleeding symptoms or abnormal categorical laboratory test results and for some numerical laboratory test results, if outside a gender-specific reference interval. For the remaining laboratory test results, suggested HPO terms were presented for manual confirmation. This was necessary to prevent inappropriate coding if a laboratory test result was abnormal because of a co-morbidity or if the reference interval was not applicable because the case was a child or pregnant. HPO terms for other clinical or laboratory abnormalities could be selected through expansion of parent HPO terms or by searching for a specific HPO term.

### Measuring similarity and clustering strength

We define the relative information content (IC) of HPO terms on the basis of their rarity within the BPD case collection. Measures of phenotypic similarity between a pair of individuals, represented as two sets of HPO terms, are then determined by the overall rareness of the pair’s shared terms [23]. The IC of term *t* is given by

$$ \mathrm{I}\mathrm{C}(t)=- \log {p}_t, $$

where *p*_*t*_ is the frequency of the term in the BPD collection. The similarity between two terms, *s* and *t*, is defined as

$$ \mathrm{s}\mathrm{i}\mathrm{m}\left(s,t\right)=\underset{v\ \in anc(s){\displaystyle \cap }anc(t)}{ \max }IC(v), $$

where anc(*x*) denotes the ancestor terms of *x*, that is, the terms {*y*|*x* ‘*is* - *a*’ *y*}. The similarity between two cases represented as two sets of terms, *D*_*a*_ and *D*_*b*_, is given by

$$ \mathrm{s}\mathrm{i}\mathrm{m}\left({D}_a,{D}_b\right)=\frac{1}{2\left|{D}_a\right|}{\displaystyle \sum_{s\in {D}_a}}\underset{t\in {D}_b}{ \max}\mathrm{s}\mathrm{i}\mathrm{m}\left(s,t\right)+\frac{1}{2\left|{D}_b\right|}{\displaystyle \sum_{s\in {D}_b}}\underset{t\in {D}_a}{ \max}\mathrm{s}\mathrm{i}\mathrm{m}\left(s,t\right). $$

This definition ensures symmetry of the similarity measure. A scale-independent measure of phenotypic dissimilarity was also computed between two cases *a* and *b* with respect to the rest of the collection:

$$ \mathrm{dist}\left(a,b\right)=\frac{1}{2}\ {\displaystyle \sum_{i\in \left\{a,b\right\}}}\ {\displaystyle \sum_{j\ \notin \left\{a,b\right\}}}{1}_{\mathrm{sim}\left({D}_i,\ {D}_j\right)\le \mathrm{s}\mathrm{i}\mathrm{m}\left({D}_a,{D}_b\right)}, $$

where *D*_*a*_ is the set of HPO terms associated with case *a*. The rank distance among a set Z of more than two cases is given by the mean over all pairwise distances:

$$ \mathrm{dist}(Z)={\left(\begin{array}{c}\hfill \left|Z\right|\hfill \\ {}\hfill 2\hfill \end{array}\right)}^{-1}{\displaystyle \sum_{a,b\in Z,a\ne b}}\ \mathrm{dist}\left(a,b\right). $$

To evaluate the phenotypic similarity of a group of cases, Z, with respect to a containing collection, a one-tail Monte Carlo *P* value for testing whether the distance between cases in Z was less than what would be expected by chance in a similar size group was computed as:

$$ \frac{1}{\left|\mathcal{W}\right|}{\displaystyle \sum_{W\in \mathcal{W}}}{1}_{\mathrm{dist}(W)\le\ \mathrm{dist}(Z)} $$

where $\mathcal{W}$ is a set of 250,000 subsets of |*Z*| index cases drawn at random from the entire collection. Although these *P* values are marginally uniform under the null, there may be a small correlation between them because the same data are reused in each set of permutations. However, if |*Z*| is small relative to the total sample size, the correlation should be negligible.

### HPO-based clustering of cases

Unsupervised clustering of unrelated BPD cases was performed by applying the Partitioning Around Medoids (PAM) algorithm [24], to a square distance matrix *M* in which the *a*th row and *b*th column was set to -*sim*(*D*_*a*_, *D*_*b*_). Affected relatives were excluded to ensure that the ensuing clusters did not depend on enrolment patterns of the affected relatives of particular index cases. The discriminatory power of HPO nodes shared by cases within each cluster was determined by performing a Fisher exact test on the contingency table containing the number of cases inside/outside the cluster vs the number of cases with/without the HPO node. Each cluster was then summarised by the HPO node having the smallest *P* value overall and up to two nodes from other distinct lineages in the HPO graph having a *P* value smaller than 10^-3^. When there were multiple nodes within a lineage fulfilling these criteria, only the most significant node was retained.

### Whole exome sequencing and variant reporting

Genomic DNA was isolated from venous blood or saliva obtained from the cases at enrolment or from archived samples. DNA library capture was performed using ROCHE NimbleGen SeqCap EZ 64 Mb Human Exome Library version 3.0 (ROCHE NimbleGen, Inc., Madison, WI, USA). The libraries were sequenced on an Illumina Hiseq 2000 instrument (Additional file 5 [25-27]). In order to filter for technical artefacts and variants unlikely to be pathogenic, variants were excluded from further analysis if they fulfilled any of the following criteria: (1) variant allele frequency >0.1% in any of the reference cohorts (Additional file 6); (2) variant not predicted to alter protein by snpEff 3.4 [28]; (3) variant not present in other affected pedigree members recruited to BRIDGE-BPD; (4) <3 reads supporting the alternate allele; (5) allele count >1 among 20 in-house whole-genome sequenced controls; (6) overall allele count >20 including exomes from other BRIDGE projects.

### Assigning causality to rare variants in known BPD genes

In order to assist analysis of variants identified in the BPD cases, we utilised the ThromboGenomics platform version 1.0 list of 49 genes that have already been linked to human platelet or coagulation disorders (Additional file 7) [29,30]. Rare variants in ThromboGenomics genes were classified using the following definitions that are consistent with current EuroGentest guidelines for assigning pathogenicity to variants [31]: (1) Pathogenic variant (PV): a variant present in the Human Genome Mutation Database (HGMD) [32] with a matched phenotype to the BRIDGE-BPD case; (2) Likely pathogenic variant (LPV): a non-HGMD variant in a gene for which previously reported cases have a matched phenotype with the BRIDGE-BPD case; (3) Variant of unknown significance (VUS): all variants that do not fulfil the previous criteria. Variants that met the criteria for a PV or LPV in an individual case were reclassified as a VUS if there was an inconsistent relationship with phenotype and mode of inheritance in other cases within the BRIDGE-BPD collection.

## Results

### Characteristics of the study collection

**Figure 1:**
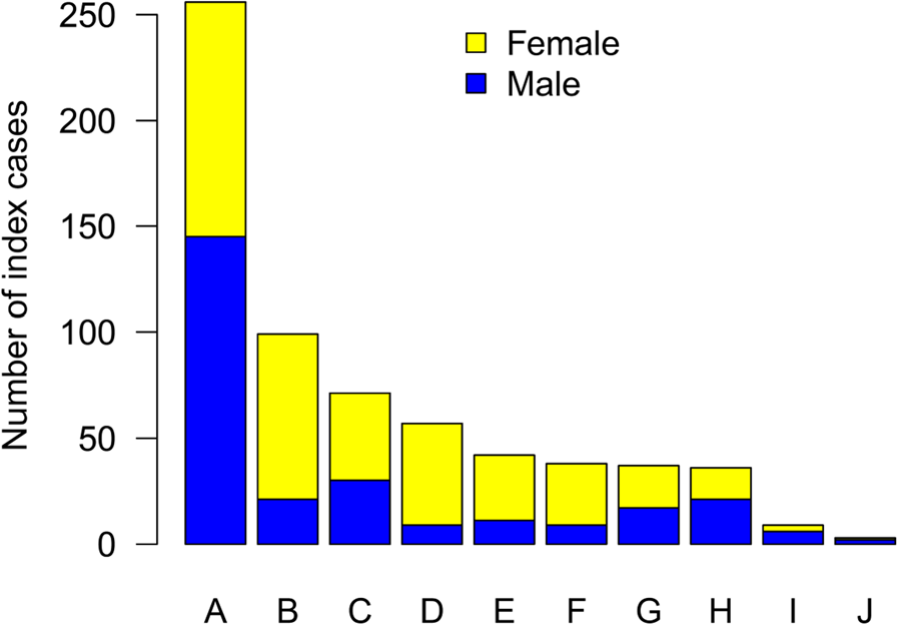
Recruitment to the BRIDGE-BPD study by enrolment centre. Gender stratified recruitment to the BRIDGE-BPD Consortium study is shown according to enrolment centre. **(A)** University of Leuven, Leuven, Belgium; **(B)** Royal Free NHS Trust, London, UK; **(C)** Centre de Référence des Pathologies Plaquettaires, Pessac, France; **(D)** Imperial College Healthcare NHS Trust, London, UK; **(E)** Cambridge University Hospitals NHS Foundation Trust, Cambridge, UK; **(F)** University Hospitals Bristol NHS Foundation Trust, Bristol, UK; **(G)** Haematological Laboratory, Trousseau Children’s Hospital and INsermU1009, Paris, France; **(H)** Children’s Hospital of Philadelphia, Philadelphia, USA; **(I)** Great Ormond Street Hospital For Children NHS Trust, London, UK; **(J)** Charité Universitätsmedizin, Berlin, Germany.

In Stage 1 of the BRIDGE-BPD study, 648 index cases and 59 affected pedigree members (414 female, 293 male) were recruited at 10 enrolment centres (Figure 1). WES has been completed for 519 cases (Additional file 8).

**Figure 2:**
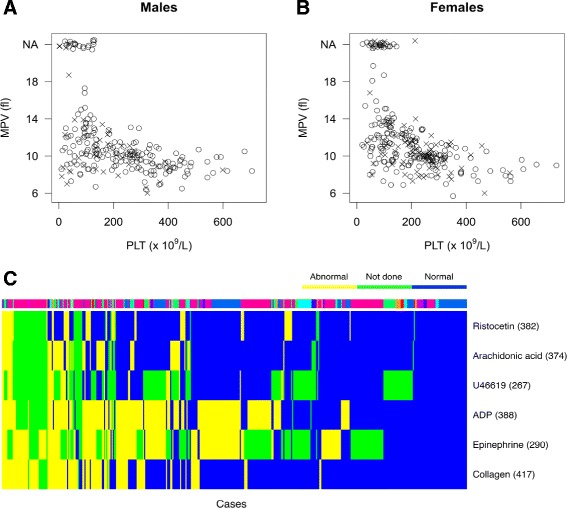
Platelet characteristics of the BRIDGE-BPD study index cases. **(A, B)** The relationship between PLT and MPV in femtolitre (fL) in male and female index cases. The crosses indicate index cases with greater than four (males) or greater than five (females) bleeding symptoms. The circles indicate index cases with fewer bleeding symptoms. NA: not available - cases in which large platelets were identified by the enrolling clinician but the MPV was not recorded. **(C)** Heat map showing the results of light transmission aggregation tests with the indicated activating agonists, classified as normal or abnormal by the enrolling clinician. The numbers in brackets indicate the number of index cases tested for each agonist. Cases are ordered by the number of abnormal or not done results across all six agonists. The bar above the heat map is colour-coded to indicate the enrolment centre for each case.

Platelet count (PLT) was recorded for 639 of the 648 index cases, of which 140 (21.9%) had PLT <100 × 10^9^/L and 53 (8.3%) had PLT >400 × 10^9^/L. Mean platelet volume (MPV) was recorded for 460 index cases of which one (0.2%) had MPV <6 fL and 91 (19.8%) had MPV >12 fL. Since some automated cytometers do not enumerate the MPV in blood samples with large platelets, enrolling clinicians were able to record whether large platelets were visible by light or electron microscopy. This identified a further 56 index cases with large platelets. Bleeding occurred across the range of platelet counts and volumes (Figure 2A,B). A total of 330 (50.9%) index cases had abnormal platelet morphology, defined as abnormal structure or size determined by light or electron microscopy (Additional file 9).

After excluding cases with PLT <100 × 10^9^/L or an unmeasured PLT in which reliability of LTA results cannot be guaranteed, there were 428 (66.0%) index cases with an LTA test result recorded for at least one agonist. A total of 196/428 (45.8%) index cases had two or more and 80/428 (18.7%) had three or more agonist responses that were classified as abnormal by the enrolling clinician (Figure 2C).

### Clinical phenotype and HPO coding

**Figure 3:**
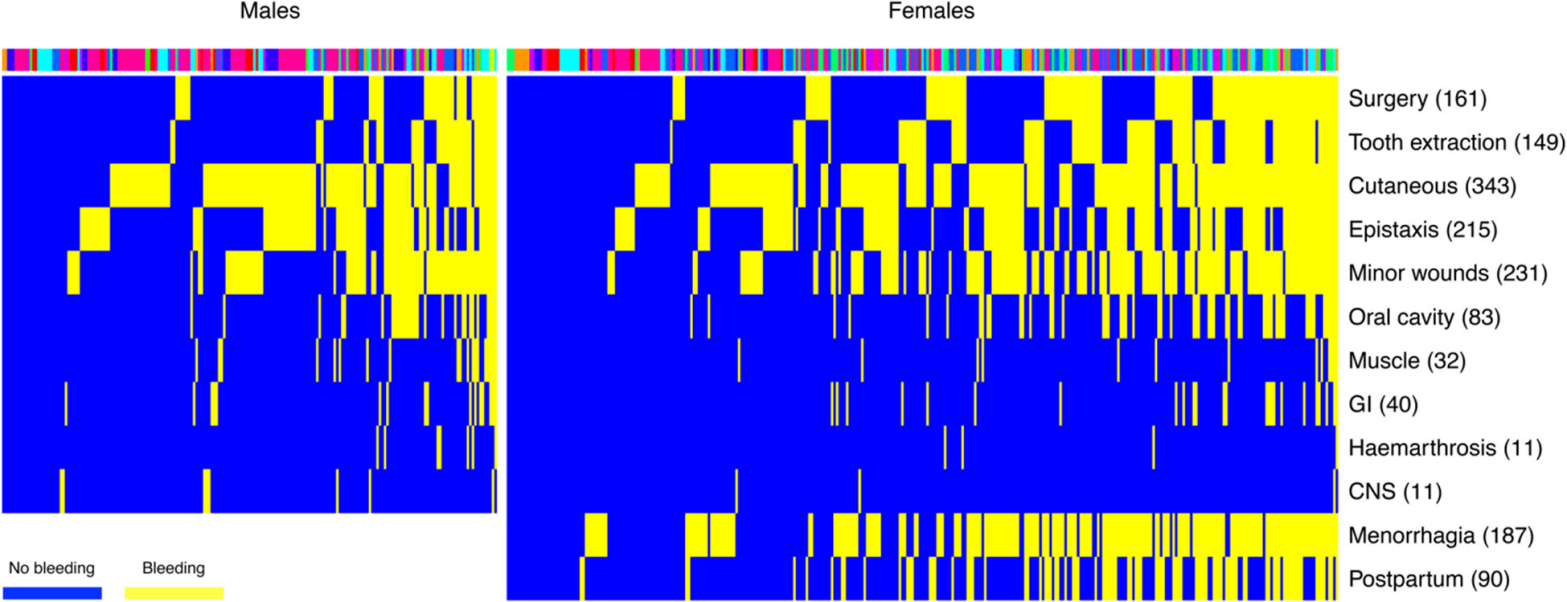
Bleeding symptoms of BRIDGE-BPD study index cases. Heat map showing the bleeding symptoms recorded for the BRIDGE-BPD index cases. The numbers in brackets indicate the number of index cases reporting each bleeding symptom. Cases are ordered by the number of bleeding symptoms. The bar above heat map is colour-coded to indicate the enrolment centre for each case.

The presence or absence of up to 10 bleeding symptoms for males and 12 bleeding symptoms for females was recorded for 528 index cases. Among the 197 male cases, 45 experienced four or more different bleeding symptoms and 12 had six or more symptoms. Among the 331 female cases, 58 experienced six or more different bleeding symptoms and nine had eight or more symptoms. The most common bleeding symptoms were cutaneous bleeding (343 index cases), bleeding from minor wounds (231 index cases) and epistaxis (215 index cases; Figure 3).

**Figure 4:**
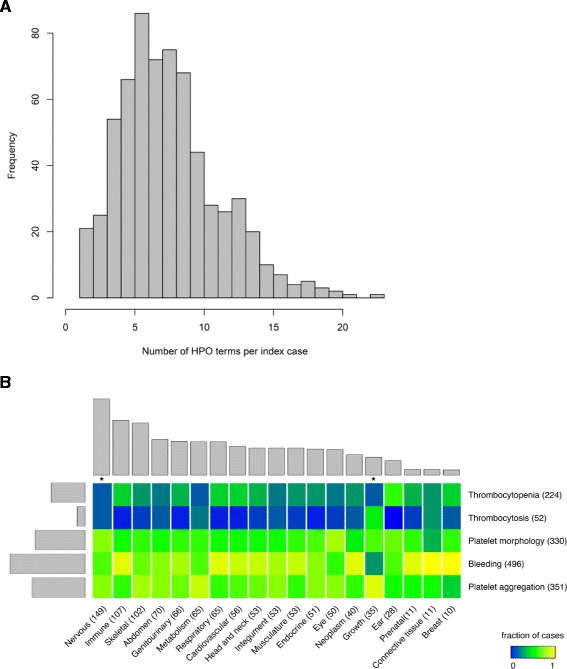
HPO terms coded in BRIDGE-BPD study index cases. **(A)** Bar plot indicating the number of human phenotype ontology (HPO) terms annotated to the index cases. **(B)** Heat map showing the relative frequencies of HPO terms pertinent to abnormalities in platelets and bleeding symptoms and in other organ or disease areas in the BRIDGE-BPD index cases. The numbers in the brackets and the barplots indicate the number of index cases with at least one HPO term pertinent to abnormality in the organ or disease area after removal of overlapping terms. *indicates that the distribution of terms pertinent to enrolment for a particular column is significantly different compared to the sum (along rows) of all other columns (*P* value <0.05 after Bonferroni correction by chi-squared test). The columns are ordered by the number of cases having a term in each leading class.

The median number of HPO terms annotated to each case was 7.5 (range 1 to 23; Figure 4A). All index cases were annotated with one or more HPO term from the ‘abnormality of blood and blood-forming tissue’ leading class. Of the 648 index cases, 387 (59.7%) were also annotated with one or more HPO term outside of ‘abnormality of blood and blood-forming tissue’ after excluding terms that are ‘descendants’ of both the ‘abnormality of blood and blood-forming tissue’ class and other leading classes (Additional file 4 and Figure 4B). The most common other HPO terms described abnormalities of the nervous system (149 index cases), immune system (107 index cases) and skeletal system (102 index cases). When index cases were grouped by abnormality in each other organ system, the frequencies of terms relevant to thrombocytopenia, thrombocytosis, bleeding, platelet morphology and platelet aggregation reflected the overall proportions of these terms in the collection for most organ systems. However, in cases with terms relevant to the nervous system and growth these terms showed significantly different distributions (*P* <0.05 after Bonferroni correction, which allows for dependence between tests in addition to multiplicity; Figure 4B).

### Clustering of cases using HPO

**Figure 5:**
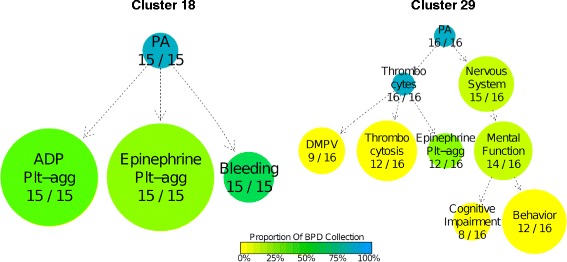
Phenotype clusters 18 and 29. Illustrative subgraphs of the HPO showing terms for the phenotype clusters 18 (15 cases) and 29 (16 cases). Arrows indicate direct (solid) or indirect (dashed) *is a* relations between terms in the ontology. DMPV: decreased mean platelet volume; PA: phenotypic abnormality; Plt-agg: platelet aggregation abnormality.

We performed unsupervised clustering of the HPO-encoded phenotype data in order to obtain an undirected characterisation of different subgroups within the heterogeneous BPD collection and assess whether particular sets of HPO terms tended to co-occur among cases in these groups. The 648 unrelated index cases were partitioned into 30 clusters ranging in size from five to 36 cases. Clinically recognisable subgroups included ‘Impaired epinephrine-induced platelet aggregation, Impaired ADP-induced platelet aggregation’ (Additional file 10, cluster 18 and Figure 5) which is a frequently reported pattern of abnormality in light transmission aggregation results identified previously as a Gi-pathway defect [33]. A further prominent cluster was ‘Autism spectrum disorder, Thrombocytosis, Decreased mean platelet volume’ (Additional file 10, cluster 29 and Figure 5) illustrating an increasingly recognised association between platelet abnormalities and neurological disorders [34].

### A new approach to gene discovery using HPO-encoded phenotype data

**Figure 6:**
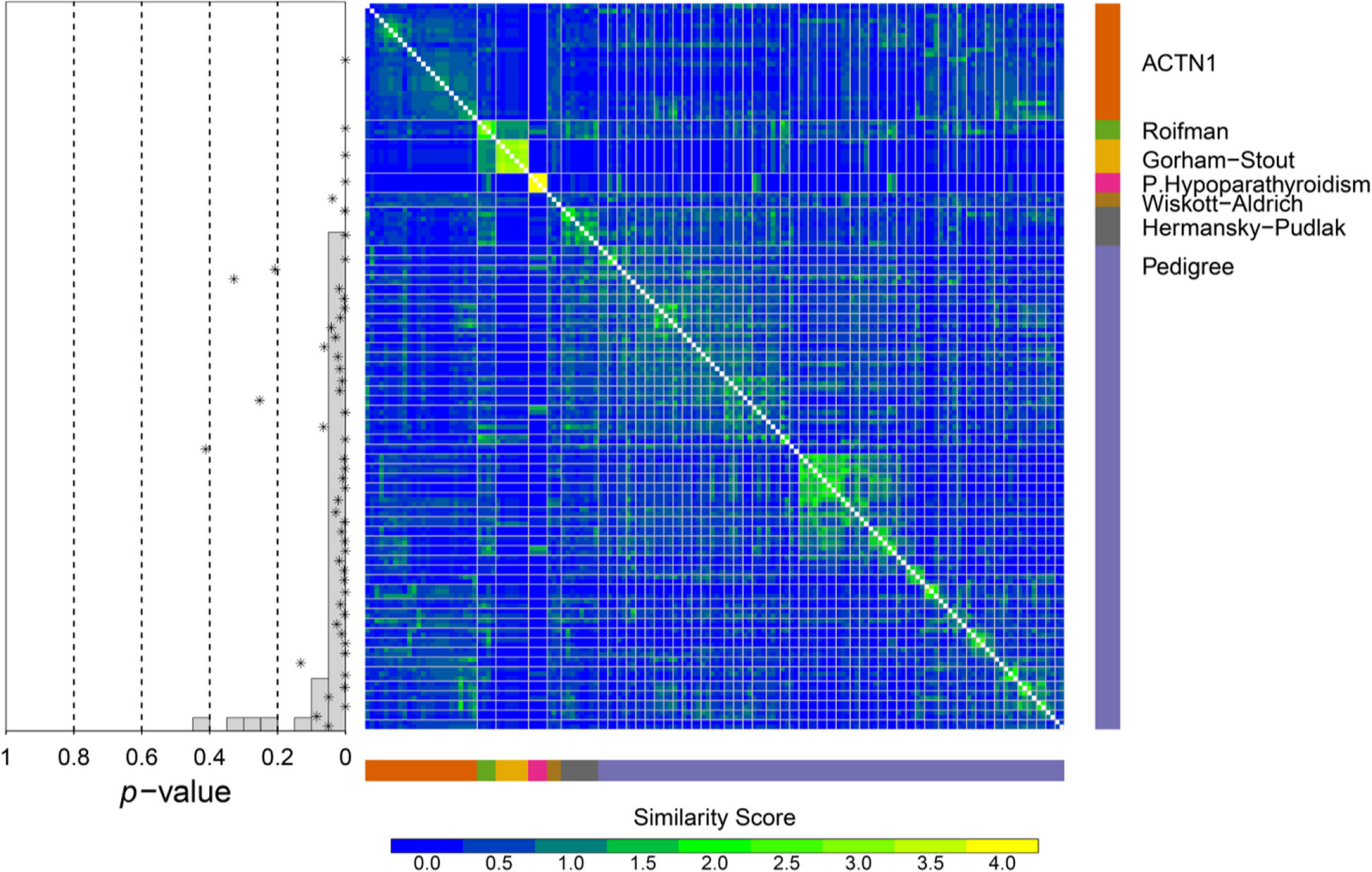
Phenotype similarity clustering of cases according to HPO terms. Heat map showing pairwise phenotypic similarity among affected members of pedigrees, cases with classical syndromes and cases with variants in *ACTN1*. The groups are ordered through complete-linkage hierarchical clustering within each class and *P* values of phenotypic similarity are shown in a scatterplot superimposed over a histogram showing the distribution of *P* values.

In order to resolve causal gene variants from the numerous candidates in a rare disease study in which genetic heterogeneity is expected, it is typically assumed that cases with causal variants in a gene or set of related genes have similar phenotypes. We therefore hypothesised that cases with rare coding variants in a disease gene would tend to cluster strongly on the basis of their HPO-encoded phenotypes. To validate this approach, phenotype closeness was first computed within the pedigree groups without provisional syndromic diagnoses recruited to the BRIDGE-BPD study, who are expected to share a causal gene variant. The phenotypic similarity *P* values corresponding to the 50 pedigrees were significantly enriched near zero (40 had *P* <0.05 (2.5 expected under the null), Figure 6). Only two pedigrees clearly failed to cluster (*P* = 0.33 and *P* = 0.42). Additionally, we validated our approach by assessing the phenotypic closeness of groups of index cases with provisional syndromic diagnoses made through clinical evaluation, as these are likely to have pertinent variants in a set of related genes. Index cases enrolled with provisional clinical diagnoses of Gorham-Stout syndrome (n = 7) (ORPHA73), HPS (n = 8), pseudohypoparathyroidism type Ib (n = 4) (ORPHA94089), Roifman syndrome (n = 4) (ORPHA353298) and WAS (n = 3) also demonstrated significantly close clustering (*P* <0.05 for each test; Figure 6). A meta-analysis using Fisher’s method to assess whether these groups clustered closely together as a whole yielded a *P* value that is less than the numerical resolution of the analysis software. These data demonstrate that despite occasional anomalies, BPD cases in which a common genetic basis is likely tend to cluster on the basis of their HPO terms.

### Cases with variants in *ACTN1* cluster strongly on the basis of their HPO terms

We applied our approach under an autosomal dominant model and discovered that the gene for which cases had the strongest phenotype similarity was *ACTN1* (*P* = 3.4 × 10^-8^). Causal variants in *ACTN1* have been identified in 18 recently reported pedigrees with a phenotype that comprises mild bleeding and macrothrombocytopenia with no other syndromic features [35-37]. Consistent with these previous descriptions, amongst the 21 index cases and three pedigree members with *ACTN1* variants in the BRIDGE-BPD analysis, 23/24 (96%) displayed thrombocytopenia and 22/24 (92%) large platelets (Table 2). HPO-based clustering of this group occurred irrespective of whether the *ACTN1* variants were classified as PV, LPV or VUS. Cluster 1 (Additional file 10) was significantly enriched for variants in *ACTN1* (Fisher’s exact test *P* value 1.79 × 10^-5^).

**Table 2:** **Rare variants identified in** ***ACTN1***

**Case**	**Transcript variant ENST00000394419**	**Protein variant ENSP00000377941.4**	**HGMD variant**	**Classification**	**PLT, ×10** ^**9**^ **/L**	**MPV, fL, and/or presence of macrothrombocytes**	**Bleeding phenotype**
B200726	14:69392385 A/C	F37C	No	LPV	57	18.1, macrothrombocytes	None
B200207	14:69392358 C/T	R46Q	Yes	PV	53	>13, macrothrombocytes	None
B200209				PV	76	>13, macrothrombocytes	Mild
B200212				PV	98	>13, macrothrombocytes	None
B200254				PV	34	>13, macrothrombocytes	None
B200735				PV	52	12.0, macrothrombocytes	None
B200746	14:69392359 G/A	R46W	No	LPV	96	15.2, macrothrombocytes	None
B200197	14:69392344 G/C	Q51E	No	LPV	113	>13, macrothrombocytes	Mild
B200836	14:69387750 C/T	V105I	Yes	PV	53	NA, macrothrombocytes	None
B200837^a^				PV	75	NA, macrothrombocytes	None
B200671	14:69371375 C/T	E225K	Yes	PV	97	13.7, macrothrombocytes	Mild
B200716				PV	82	15.0, macrothrombocytes	None
B200398	14:69369274 C/T	V228I	No	LPV	31	15.4, macrothrombocytes	Mild
B200280	14:69358897 C/T	R320Q	No	LPV	108	15.1, macrothrombocytes	Mild
B200281^a^				LPV	111	13.9, macrothrombocytes	None
B200835	14:69352254 C/T	A425T	No	VUS	50	10.0, no macrothrombocytes	Mild
B200283	14:69349768 A/G	L547P	No	LPV	91	13.3, macrothrombocytes	Mild
B200048	14:69349648 G/A	A587V	No	VUS	390	NA, no macrothrombocytes	Mild
B200284	14:69346749 G/T	T737N	No	LPV	60	16.1, macrothrombocytes	Mild
B200285^a^				LPV	48	16.8, macrothrombocytes	Mild
B200741	14:69346747 G/A	R738W	Yes	PV	94	12.9, macrothrombocytes	None
B200745				PV	70	14.5, macrothrombocytes	None
B200750	14:69346746 C/T	R738Q	No	LPV	106	14.0, macrothrombocytes	None
B200414	14:69346704 C/G	R752P	No	LPV	121	11.4, macrothrombocytes	Mild

^a^Affected family member.

### Pertinent and non-pertinent rare variants in *MYH9*

We also assessed the HTS and data analysis pipelines with the autosomal dominant disorder *MYH9*-RD. We selected this disorder because previous descriptions of *MYH9*-RD suggested that cases are usually readily identified clinically and were unlikely to have been recruited. Despite this, we identified nine different *MYH9* missense variants in 13 unrelated BPD cases and one pedigree case. These included seven index cases with rare variants classified as PV or LPV and six index cases with VUS. In order to investigate whether HPO annotation and cluster analysis would have enabled detection of these cases as a distinct phenotype group we explored the HPO terms that had been assigned to these cases. The seven index cases with PV or LPV in *MYH9* included six cases with terms indicating macrothrombocytes, six with terms indicating thrombocytopenia and one with neutrophil Döhle-bodies but none with other recognised features such as renal impairment, sensorineural deafness or cataract (Table 3). The seven index cases with PV or LPV clustered closely together based on the HPO terms (*P* = 0.005) while the six index cases with VUS did not cluster (*P* = 0.684). However, the 13 index cases as a whole did not cluster strongly based on their HPO phenotypes (*P* = 0.363) due to the dilution of PV with VUS. This observation highlights the need for improved algorithms for selecting candidate variants based on predictions of pathogenicity but also supports our stringent approach for assigning causality to variants in known genes.

**Table 3:** **Rare variants identified in** ***MYH9*** **and validated by Sanger sequencing**

**Case**	**Transcript variant ENST00000216181**	**Protein variant ENSP00000216181**	**HGMD variant**	**Classification**	**PLT, ×10** ^**9**^ **/L**	**MPV, fL and/or presence of macrothrombocytes**	**Other** ***MYH9*** **-RD characteristics**
B200760	22:36744995 G/A	S96L	Yes	PV	180	Macrothrombocytes	None
B200771	22:36705438 C/A	D578Y	No	VUS	184	10.1	None
B200423	22:36696237 G/A	A971V	No	VUS	262	10.2	None
B200024	22:36691696 A/G	S1114P	Yes	VUS	164	NA	None
B200245				VUS	53	11.1, Macrothrombocytes	None
B200243	22:36691115 G/A	R1165C	Yes	PV	22	Macrothrombocytes	None
B200594				PV	46	Macrothrombocytes	None
B200595^a^				PV	61	Macrothrombocytes	None
B200614	22:36688151 C/T	D1409N	No	VUS	319	9.8	None
B200752				VUS	149	10.1, Macrothrombocytes	None
B200855				VUS	95	16.8, Macrothrombocytes	None
B200208	22:36688106 C/T	D1424N	Yes	PV	99	13.6	None
B200010	22:36685249 G/C	S1480W	No	VUS	244	NA	None
B200244	22:36678800 G/A	R1933X	Yes	PV	26	Macrothrombocytes	Döhle inclusions

Other MYH9-RD characteristics sought were the presence of Döhle inclusions, cataract, deafness or renal pathology.

^a^Father of B200594.

### Variant identification in the ThromboGenomics gene list

The BRIDGE-BPD HTS and data analysis pipelines were tested by evaluating coding variants in the ThromboGenomics list of known genes linked to autosomal recessive or X-linked recessive BPD. Since cases with BPD of known genetic aetiology were excluded from enrolment, we predicted that causal variants in the ThromboGenomics genes would be uncommon. Consistent with this, we identified PV in only two ThromboGenomics genes that completely explained the phenotype of one case with HPS3 and two cases with WAS. Further cases with PV in *F9* and in *F8* displayed reduced FIX and FVIII activity, respectively, that were explained by the observed PV. However, these cases also displayed abnormal platelet function indicating that the PV only partially explained the phenotype. Three cases had variants classified as LPV in ThromboGenomics genes, which cannot be considered causal for the BPD phenotype without further confirmatory investigations (Table 4). A further 10 index cases had variants classified as VUS in ThromboGenomics genes because there was no plausible association between the gene and phenotype (Additional file 11). This group included cases with variants in coagulation factor genes who had normal levels of coagulation factors.

**Table 4:** **Pathogenic and likely pathogenic variants identified in genes associated with autosomal recessive and X-linked recessive bleeding and platelet disorders**

**Case**	**Position**	**Gene**	**Ref**	**Alt**	**Genotype**	**HGMD**	**Effect** ^**a**^	**Haematological HPO terms**	**Other HPO terms**	**Classification:**	
										**Variant**	**Phenotype**
B200286	3:148881737	*HPS3*	G	C	C|C	Yes	Abnormal splicing	Bleeding with minor or no trauma, subcutaneous haemorrhage, menorrhagia, postpartum haemorrhage, impaired ADP-induced platelet aggregation, impaired epinephrine-induced platelet aggregation, epistaxis, prolonged bleeding after surgery, prolonged bleeding after dental extraction, increased mean platelet volume.	Hypothyroidism, visual impairment, nystagmus, albinism.	PV	Explained
B200412	3:148858819	*HPS3*	T	TA	T|TA	No	Frameshift	Impaired epinephrine-induced platelet aggregation, bleeding with minor or no trauma, subcutaneous haemorrhage, epistaxis, menorrhagia, prolonged bleeding after surgery, abnormal dense granules.	Ocular albinism.	LPV	Possibly explained
	3:148876539	*HPS3*	G	A	G|A	No	W593^a^			LPV	
B200068	10:103827041	*HPS6*	C	G	C|G	No	L604V	Increased mean platelet volume.	Congenital cataract, strabismus, maternal diabetes.	LPV	Possibly explained
	10:103827554	*HPS6*	C	G	C|G	No	L775V			LPV	
B200196	X:48542673	*WAS*	C	T	T	Yes	T45M	Thrombocytopenia, abnormal bleeding, decreased mean platelet volume, abnormal platelet shape.	Recurrent infections.	PV	Explained
B200725	X:48544145	*WAS*	T	C	C	Yes	F128S	Monocytosis, neutrophilia, thrombocytopenia, leukocytosis, subcutaneous haemorrhage, gastrointestinal haemorrhage.		PV	Explained
B200443	X:138633272	*F9*	G	A	A	Yes	R191H	Reduced factor IX activity, impaired ADP-induced platelet aggregation, bleeding with minor or no trauma, spontaneous haematomas, abnormal number of dense granules.		PV	Partially explained
B200452	X:154124407	*F8*	C	G	G	Yes	S2125T	Reduced factor VIII activity, persistent bleeding after trauma, prolonged bleeding after surgery, prolonged bleeding after dental extraction, bleeding requiring red cell transfusion, impaired collagen-induced platelet aggregation, bleeding with minor or no trauma, joint haemorrhage, abnormal platelet shape, abnormal number of dense granules.		PV	Partially explained
B200772	X:154176011	*F8*	A	G	G	No	F692S	Reduced factor VIII activity, bruising susceptibility, impaired ADP-induced platelet aggregation, impaired collagen-induced platelet aggregation, impaired thromboxane A2 agonist-induced platelet aggregation, impaired ristocetin-induced platelet aggregation, impaired arachidonic acid-induced platelet aggregation, impaired thrombin-induced platelet aggregation, abnormal platelet granules, bleeding with minor or no trauma.		LPV	Possibly partially explained

Alt: alternative; Ref: reference.

^a^Effect considered relative to the Consensus Coding Sequence (CCDS) for each gene.

## Discussion

Heritable BPD are individually rare but collectively common diseases that have heterogeneous clinical characteristics. This clinical complexity, genetic heterogeneity and the large number of candidate genes for BPD has previously hampered gene discovery. In this exploratory Stage 1 of the BRIDGE-BPD study, we first enhanced HPO terminology to enable standardized annotation of the phenotypes of BPD cases. After enrolling the largest collection of BPD cases reported to date, we demonstrated that HPO annotation enabled the characterisation of clusters of BPD cases with similar phenotypes, which we hypothesise have causal genetic variants in the same or related genes. The use of HPO to facilitate research within neurogenetics by comparing standard database descriptions of diseases has been described previously [18]. However, this is the first report of the application of HPO to characterise individual case phenotypes and to aid gene discovery through statistical cluster analysis.

The complex clinical characteristics of the 648 index cases in the Stage 1 BRIDGE-BPD study reflect our desire to enrol a comprehensive collection of cases with different subgroups of disorders within BPD, including bleeding of unknown origin. Previously reported collections have comprised BPD cases without prior investigation [4,5] and collections linked by similar phenotypes such as abnormal platelet number [38], platelet function [33] or von Willebrand disease [21,39]. Thus, the BRIDGE-BPD collection is unique in its size and diversity. In common with previous collections, the BRIDGE-BPD cases were predominantly females, who experienced more bleeding symptoms than males because of obstetric and heavy menstrual bleeding. There was marked heterogeneity in PLT, MPV, platelet morphology, and particularly in LTA platelet function test results, highlighting the diversity of heritable platelet function disorders [33].

A striking finding from the Stage 1 BRIDGE-BPD study was that a median of 7.5 HPO terms were required to annotate the phenotype of each case. These comprised at least one term from ‘abnormality of blood and blood-forming tissue’, reflecting the study inclusion criteria. However, 60% of cases also had HPO terms reflecting abnormality in other organ systems, even after removing overlapping terms. We also observed a statistically significant difference in the frequency of five haematological HPO terms in cases with a nervous system abnormality compared to other cases (*P* = 0.001) reflecting more frequent platelet function and morphology defects. This is consistent with the existing literature indicating that neurological and platelet function disorders often coincide [34]. The statistically significant difference in the frequency of the five haematological HPO terms between cases with abnormal growth and those without (*P* = 8.12 × 10^-6^) arose because of enrolment of a group of cases with growth defects at a single centre. One potential criticism of Stage 1 of the BRIDGE-BPD study is that cases were recruited from some centres with highly specialist interests, which may reduce the wider applicability of the study findings. This effect is illustrated by our finding of phenotypic associations between haematological terms and both growth and neurological terms arising from the specialist practice at the University of Leuven. However, this effect is partially offset by recruitment from the UK enrolment centres which are more typical tertiary referral centres, and will not occur in subsequent stages of the BRIDGE-BPD project which will recruit more widely.

A further unique attribute of the BRIDGE-BPD methodology is that it enables calculation of the closeness between groups of HPO terms annotated to different cases. This provides a measure of phenotypic similarity between cases and enables the definition of case subgroups to assist the analysis of genotype data. We have provided proof of principle of this approach by demonstrating low *P* values for phenotypic similarity between BPD cases from the same pedigrees and between unrelated cases with provisional clinical diagnoses of syndromic BPD, in which both groups are likely to share a causal genetic variant. This included examples with known candidate genes (HPS and WAS) and with unknown genes (Gorham-Stout and Roifman syndromes, pseudohypoparathyroidism type Ib).

We also evaluated phenotype similarity in groups of cases sharing a rare coding variant in the same gene. The most significantly similar group corresponded to cases harbouring variants in *ACTN1*. Although this particular association could be found through traditional regression-based approaches against platelet count, the HPO approach allows associations between genotypes and combinations of any kind of HPO-encoded phenotypes to be discerned. Here, an association was found between the presence of a variant and two terms jointly - the ‘Thrombocytopenia’ and ‘Increased Mean Platelet Volume’ terms - while regression analysis is typically performed trait by trait in a univariate fashion. Certainly, standard regression methods cannot be used to model heterogeneous combinations of data types (binary, quantitative, categorical), nor can they account for the hierarchical nature of ontologically encoded outcome data. Furthermore, patient HPO data can be linked to data in online human databases such as OMIM, and to model system databases such as the Mouse Phenotype Ontology. In future, this could be used to prioritise genes based on the similarity of groups of cases to ontological phenotype terms derived from the literature.

We assessed index cases harbouring variants in *MHY9* who were not considered likely to have MYH9-RD by the enrolling clinicians based on clinical evaluation. In this group, the cases with PV or LPV in *MYH9* displayed a high degree of similarity whereas individuals with VUS in *MYH9* were not similar. Furthermore, the index cases with PV or LPV in *MYH9* were phenotypically different from the index cases with VUS. This illustrates that HPO-based clustering can reveal phenotype and genotype links that cannot be made by enrolling clinicians in isolation but only in combination with powerful methods for distinguishing variants by predicted pathogenicity.

We also demonstrated potential limitations of this approach arising from differences in phenotype coding between and within enrolling centres. For example, two pedigrees showed poor clustering by phenotype, with phenotypic similarity *P* values of 0.42 and 0.33. In one of these pedigrees, the lack of similarity between the two members was because the index case had detailed phenotypic evaluation but the pedigree case consented only to limited evaluation. In the other pedigree, the lack of similarity was due to the marked difference in age at evaluation. This illustrates how the age-dependence of HPO terms such as bleeding symptoms may result in paediatric cases clustering away from adult cases. The potential confounding effect caused by gender-specific HPO terms was minimised by excluding these terms from calculation of similarity scores. Despite these limitations, recruiting from a heterogeneous set of centres is crucial to obtain the sample sizes required to achieve power for gene discovery.

The approach we have presented for computing similarity scores relies on pre-selecting groups of cases based on the presence of rare variants fulfilling certain criteria, such as presumed mode of inheritance. Sometimes, only a subset of the cases in a group will be explained by variants in the same gene, thus diluting the strength of phenotypic similarity of the entire group. For *ACTN1*, which was used to model similarity clustering analysis, the effect of the dilution was minimal, but in other cases it may play a larger role. The development of new methods to offset this effect is a worthwhile area of future research.

## Conclusions

We have demonstrated that HPO annotation of the large and diverse BRIDGE-BPD collection enables the identification of clusters of individuals with phenotypic similarities who are likely to have causal genetic variants in the same or related genes. Our international consortium has enabled comparison of standardised phenotypic descriptions among cases across the world, which improves statistical power by increasing the size of case groups.

We have validated the methodologies developed for the BRIDGE-BPD study, using BPD such as *ACTN1*-related disorder, which have been newly reported since the start of recruitment to our study collection. However, these approaches are equally applicable to disorders of unknown genetic basis, in which cases are also expected to show similarity of HPO terms. It is noteworthy that the cases in the stage 1 BRIDGE-BPD study with PV or LPV in established BPD genes together account for less than 10% of the study collection. We anticipate that identification of further case subgroups based on similarity of HPO terms will be a powerful source of new gene discoveries in the remainder of the study collection and among cases in the ongoing enrolment programme. This gene discovery approach, pioneered here in bleeding and platelet disorders, is broadly applicable to other rare disease groups.

## Additional files

Additional file 1:
**A table listing national ethics authorities and study approval numbers.**
Additional file 2:
**A table containing the bleeding symptoms recorded in the BRIDGE-BPD study.**
Additional file 3:
**A table indicating the HPO terms added to enable annotation of bleeding and platelet phenotypes.**
Additional file 4:
**A figure demonstrating the overlap of HPO terms between the ‘abnormality of the blood and blood-forming tissues’ and the other leading classes.**
Additional file 5:
**A description of the whole exome sequencing methodology used in the BRIDGE-BPD study.**
Additional file 6:
**A table containing details of the reference cohorts used in this study.**
Additional file 7:
**A table listing the ThromboGenomics genes.**
Additional file 8:
**A table indicating recruitment to the BRIDGE-BPD study at the completion of Stage 1.**
Additional file 9:
**A figure showing the HPO terms used to describe abnormal platelet morphology.**
Additional file 10:
**A table listing 30 phenotype clusters identified in the BRIDGE-BPD cohort.**
Additional file 11:
**A table listing variants of uncertain significance identified in genes associated with autosomal recessive and X-linked recessive bleeding and platelet disorders.**
Additional file 12:
**A table listing additional members of the BRIDGE-BPD Consortium.**

## Abbreviations

BPD: bleeding and platelet disorders
HGMD: Human Genome Mutation Database
HPO: Human Phenotype Ontology
HPS: Hermansky-Pudlak syndrome
HTS: high throughput sequencing
IC: information content
ICD: International Classification of Diseases
LPV: likely pathogenic variant
LTA: light transmission aggregometry
MPV: mean platelet volume
*MYH9*-RD: *MYH9*-related disorder
OMIM: Online Mendelian Inheritance in Man
PAM: Partitioning Around Medoids
PLT: platelet
PV: pathogenic variant
SNOMED-CT: Systematized Nomenclature of Medicine Clinical Terminology
VUS: variant of unknown significance
WAS: Wiskott-Aldrich syndrome
