# Supplementary material for: Human phenotype ontology annotation and cluster analysis to unravel genetic defects in 707 cases with unexplained bleeding and platelet disorders
Source: Genome Med. 2015 Apr 9;7(1):36. doi: 10.1186/s13073-015-0151-5 (PMC4422517; doi:10.1186/s13073-015-0151-5)
Supplement: Additional file 1: — A table listing national ethics authorities and study approval numbers. [file 13073_2015_151_MOESM1_ESM.pdf]

Additional file 1. National ethics authorities and approval numbers

Name of national ethics authority responsible	Ethics approval number	Country
Cambridgeshire 1 Research Ethics Committee	10/H0304/66	United Kingdom
Institut National de La Santé et de la Recherche Médicale	RBM-01-14	France
Sir Charles Gairdner Group Human Research Ethics Committee	2012-095	Australia
Ethics Committee of the University Hospital Leuven	ML3580	Belgium
Ethics board of the University of Greifswald	n/a	Germany
Ethics Board 2 at Campus Virchow-Klinikum, Charité University Hospital, Berlin	EA2/170/05	Germany
Children’s Hospital of Philadelphia Institutional Review Board	IRB#12-008603	USA
Beth Israel Deaconess Medical Center IRB	Protocol #: 2011P000337	USA
