# Supplementary material for: Human phenotype ontology annotation and cluster analysis to unravel genetic defects in 707 cases with unexplained bleeding and platelet disorders
Source: Genome Med. 2015 Apr 9;7(1):36. doi: 10.1186/s13073-015-0151-5 (PMC4422517; doi:10.1186/s13073-015-0151-5)
Supplement: Additional file 2: — A table containing the bleeding symptoms recorded in the BRIDGE-BPD study. [file 13073_2015_151_MOESM2_ESM.pdf]

Additional file 2: Bleeding symptoms recorded in the BRIDGE-BPD study.

**All cases**
Epistaxis
Cutaneous
Bleeding from minor wounds
Oral cavity
Tooth extraction
Surgery
Muscle hematomas
Hemarthrosis
Central nervous system bleeding
Gastrointestinal bleeding
**Female cases only**
Menorrhagia
Post-partum hemorrhage
