# Supplementary material for: Human phenotype ontology annotation and cluster analysis to unravel genetic defects in 707 cases with unexplained bleeding and platelet disorders
Source: Genome Med. 2015 Apr 9;7(1):36. doi: 10.1186/s13073-015-0151-5 (PMC4422517; doi:10.1186/s13073-015-0151-5)
Supplement: Additional file 3: — A table indicating the HPO terms added to enable annotation of bleeding and platelet phenotypes. [file 13073_2015_151_MOESM3_ESM.pdf]

**Additional file 3: HPO terms added to enable annotation of bleeding and platelet phenotypes.**

| HPO ID | HPO Term |
|---|---|
| HP:0001973 | Autoimmune thrombocytopenia |
| HP:0003540 | Impaired platelet aggregation |
| HP:0005527 | Reduced kininogen activity |
| HP:0005528 | Bone marrow hypocellularity |
| HP:0005561 | Abnormality of bone marrow cell morphology |
| HP:0008148 | Impaired epinephrine-induced platelet aggregation |
| HP:0011870 | Impaired arachidonic acid-induced platelet aggregation |
| HP:0011871 | Impaired ristocetin-induced platelet aggregation |
| HP:0011872 | Impaired thrombin-induced platelet aggregation |
| HP:0011877 | Increased mean platelet volume |
| HP:0011878 | Abnormal platelet membrane protein expression |
| HP:0011879 | Decreased platelet glycoprotein Ib-IX-V |
| HP:0011881 | Decreased platelet glycoprotein VI |
| HP:0011882 | Decreased platelet P2Y12 receptor |
| HP:0011883 | Abnormal platelet granules |
| HP:0011894 | Impaired thromboxane A2 agonist-induced platelet aggregation |
| HP:0012483 | Abnormal alpha granules |
| HP:0012484 | Abnormal dense granules |
| HP:0012491 | Abnormal dense tubular system |
| HP:0004813 | Post-transfusion thrombocytopenia |
| HP:0002584 | Intestinal bleeding |
| HP:0007420 | Spontaneous hematomas |
| HP:0007902 | Vitreous hemorrhage |
| HP:0011884 | Abnormal umbilical stump bleeding |
| HP:0011885 | Hemorrhage of the eye |
| HP:0011888 | Bleeding requiring red cell transfusion |
| HP:0011890 | Prolonged bleeding following procedure |
| HP:0011891 | Post-partum hemorrhage |
| HP:0011895 | Anemia due to reduced life span of red cells |
| HP:0011896 | Subconjunctival hemorrhage |
| HP:0011897 | Neutrophilia |
| HP:0011898 | Abnormality of circulating fibrinogen |
| HP:0011899 | Hyperfibrinogenemia |
| HP:0011901 | Dysfibrinogenemia |
| HP:0012130 | Abnormality of cells of the erythroid lineage |
| HP:0012131 | Abnormal number of erythroid precursors |

| | |
|---|---|
| HP:0012132 | Erythroid hyperplasia |
| HP:0012133 | Erythroid hypoplasia |
| HP:0012135 | Abnormality of cells of the granulocytic lineage |
| HP:0100827 | Lymphocytosis |
| HP:0012146 | Abnormality of von Willebrand factor |
| HP:0012147 | Reduced quantity of von Willebrand factor |
| HP:0012148 | Multiple lineage myelodysplasia |
| HP:0012149 | Bilineage myelodysplasia |
| HP:0012150 | Single lineage myelodysplasia |
| HP:0008320 | Impaired collagen-induced platelet aggregation |
| HP:0011873 | Abnormal platelet count |
| HP:0011875 | Abnormal platelet morphology |
| HP:0011889 | Bleeding with no or minor trauma |
| HP:0011869 | Abnormal platelet function |
| HP:0011874 | Heparin-induced thrombocytopenia |
| HP:0011876 | Abnormal platelet volume |
| HP:0011880 | Acute disseminated intravascular coagulation |
| HP:0011886 | Hyphema |
| HP:0011887 | Choroid hemorrhage |
| HP:0011892 | Vitamin K deficiency |
| HP:0011893 | Abnormal leukocyte count |
| HP:0011900 | Hypofibrinogenemia |
| HP:0011902 | Abnormal hemoglobin |
| HP:0011903 | Hemoglobin H |
| HP:0011904 | Persistence of hemoglobin F |
| HP:0011905 | Reduced hemoglobin A |
| HP:0011906 | Reduced beta/alpha synthesis ratio |
| HP:0011907 | Reduced alpha/beta synthesis ratio |
| HP:0011908 | Unilateral radial aplasia |
| HP:0011909 | Flattened metacarpal heads |
| HP:0011910 | Shortening of all phalanges of fingers |
| HP:0011911 | Abnormality of metocarpophalangeal joint |
| HP:0003010 | Prolonged bleeding time |
| HP:0012524 | Abnormal platelet shape |
| HP:0012525 | Abnormal alpha granule distribution |
| HP:0012526 | Absence of alpha granules |
| HP:0012527 | Abnormal alpha granule content |
| HP:0012528 | Abnormal number of alpha granules |
| HP:0012529 | Abnormal dense granule content |

| | |
|---|---|
| HP:0012530 | Abnormal number of dense granules |
| HP:0011460 | Embryonal onset |
| HP:0011461 | Fetal onset |
