# Supplementary material for: Human phenotype ontology annotation and cluster analysis to unravel genetic defects in 707 cases with unexplained bleeding and platelet disorders
Source: Genome Med. 2015 Apr 9;7(1):36. doi: 10.1186/s13073-015-0151-5 (PMC4422517; doi:10.1186/s13073-015-0151-5)
Supplement: Additional file 4: — A figure demonstrating the overlap of HPO terms between the ‘abnormality of the blood and blood-forming tissues’ and the other leading classes. [file 13073_2015_151_MOESM4_ESM.pdf]

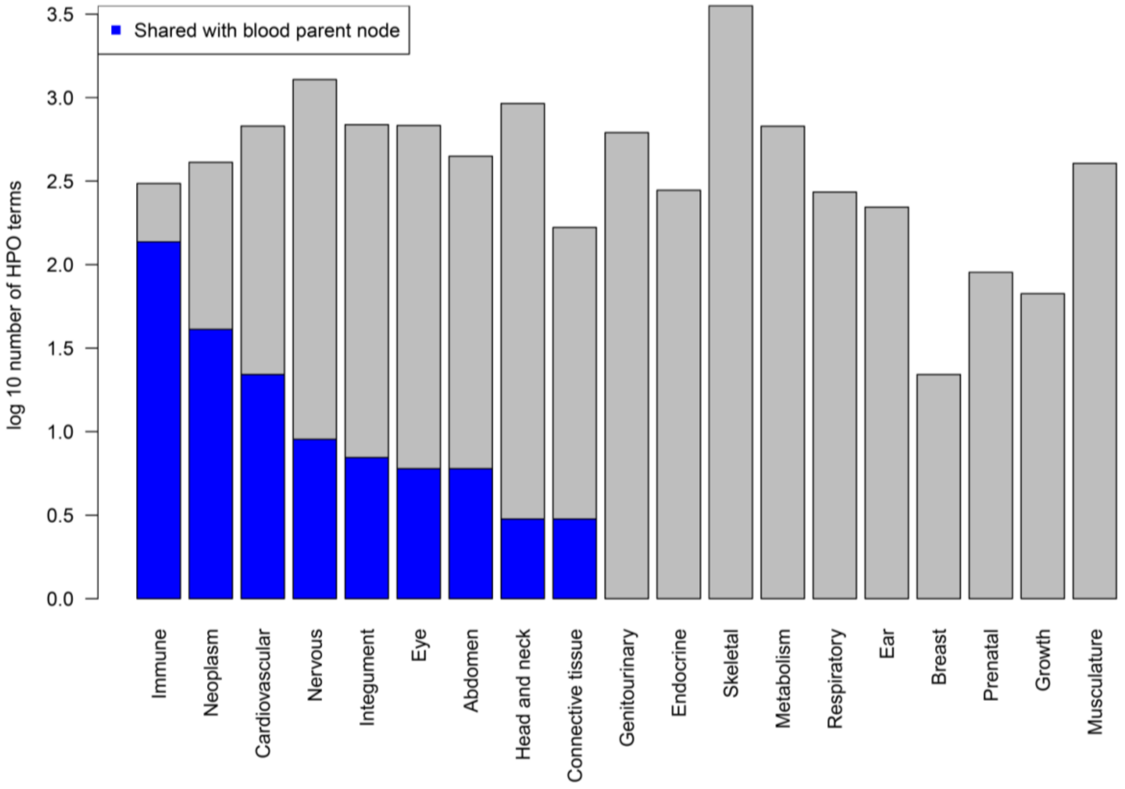

Additional file 4: Overlap of HPO terms in each leading class with the *abnormality of the blood and blood forming tissue* leading class. The bar plot depicts the number of HPO terms within each leading class and the number that are shared with the *abnormality of the blood and blood forming tissue* leading class.
