# Supplementary material for: Human phenotype ontology annotation and cluster analysis to unravel genetic defects in 707 cases with unexplained bleeding and platelet disorders
Source: Genome Med. 2015 Apr 9;7(1):36. doi: 10.1186/s13073-015-0151-5 (PMC4422517; doi:10.1186/s13073-015-0151-5)
Supplement: Additional file 5: — A description of the whole exome sequencing methodology used in the BRIDGE-BPD study. [file 13073_2015_151_MOESM5_ESM.pdf]

Human phenotype ontology annotation and cluster analysis to unravel genetic defects in 707 cases with unexplained bleeding and platelet disorders.

Additional file 5: supplementary methods

Genomic DNA was isolated from venous blood or saliva obtained from the cases at enrolment or from archived samples. Extracted DNA was quality controlled by gel electrophoresis and by three independent measurements of DNA concentration by Picogreen (Life Technologies Ltd, Paisley, UK), Qubit (Life Technologies) and Glomax (Promega, Madison, USA). DNA samples were processed in batches of 94 in order to include a positive and a negative control samples. For each case 1µg of their DNA sample was fragmented using Covaris E220 (Covaris Inc., Woburn, MA, USA) to obtain an average size of 200 base pair (bp) DNA fragments. DNA samples were processed using the Illumina TruSeq DNA LT Sample Prep kit (Illumina Inc., San Diego, CA, USA) on the Beckman Biomek FX automated workstation (Beckman Coulter Inc., Brea, CA, USA). DNA libraries were captured in pairs for three days using ROCHE NimbleGen SeqCap EZ 64Mb Human Exome Library version 3.0 (ROCHE NimbleGen, Inc. Madison, WI, USA). The final libraries were checked using LabChip (PerkinHelmer, Hopkinton, MA, USA) and Glomax for size and concentration, respectively. The success of the enrichment was tested by qPCR measuring the relative abundance of four control target regions before and after DNA capture. A second qPCR using the KAPA Library quantification kit (KAPA Biosystem, Ltd, Cape Town, South Africa) was performed to quantify the final captured libraries. Six libraries were sequenced as 100bp paired-end reads on the Illumina Hiseq 2000 instrument generating around 160-180M reads per lane. Trimming of adapter-contaminated or

poor quality sequence reads were performed as described previously[22]. Reads were aligned to the GRCh 37 build of the human reference genome using BWA 0.6.2[23]. Realignment around indels and base call quality recalibration was performed using GATK 2.3_9[24]. Likely PCR duplicates were marked with Picard 1.89 (http://picard.sourceforge.net). Samples were excluded from analysis if the coverage was below 10X in 70% of the genome or if the estimated contamination rate computed by VerifyBamId 1.0.0[25] was >5%. Variants were called in the exome capture targets and in 1000bp flanking regions using the GATK 2.8_1 Unified Genotyper.
