# Supplementary material for: Human phenotype ontology annotation and cluster analysis to unravel genetic defects in 707 cases with unexplained bleeding and platelet disorders
Source: Genome Med. 2015 Apr 9;7(1):36. doi: 10.1186/s13073-015-0151-5 (PMC4422517; doi:10.1186/s13073-015-0151-5)
Supplement: Additional file 6: — A table containing details of the reference cohorts used in this study. [file 13073_2015_151_MOESM6_ESM.pdf]

Additional file 6: Details of reference cohorts.

Cohort	Sequencing	Number of individuals	Details
1000 Genomes	Low pass, whole genome	1092	Controls of African, European and Asian ancestry from the HapMap collection.
UK10K-WES*	Whole exome	4732	Clinical cases with neurodevelopmental disorders, obesity or rare diseases.
UK10K-WGS	Low pass, whole genome	3621	Controls from the ALSPAC and TwinsUK cohorts.
Exome Sequencing Project	Whole exome	6503	2203 African-American /4300 European-American controls and cases of early onset myocardial infarction, stroke or lung disease.

* Data from this cohort were available for the following ThromboGenomics genes: *GATA1*, *GP1BA*, *GP1BB*, *GP6*, *ITGA2B*, *ITGB3*, *VWF*.
