# Supplementary material for: Human phenotype ontology annotation and cluster analysis to unravel genetic defects in 707 cases with unexplained bleeding and platelet disorders
Source: Genome Med. 2015 Apr 9;7(1):36. doi: 10.1186/s13073-015-0151-5 (PMC4422517; doi:10.1186/s13073-015-0151-5)
Supplement: Additional file 7: — A table listing the ThromboGenomics genes. [file 13073_2015_151_MOESM7_ESM.pdf]

Additional file 7: ThromboGenomics gene list.

Gene name	Disorder
P2RY12	P2Y12 defect
HOXA11	Amegakaryocytic thrombocytopenia with radio-ulnar synostosis
GP1BA	Bernard-Soulier syndrome
GP1BB	Bernard-Soulier syndrome
GP9	Bernard-Soulier syndrome
GP6	Bleeding diathesis due to glycoprotein VI deficiency
LYST	Chediak-Higashi syndrome
MPL	Congenital amegakaryocytic thrombocytopenia
RUNX1	Familial platelet disorder with predisposition to acute myelogenous leukemia
TBXAS1	Ghosal hematodiaphyseal dysplasia
ITGA2B	Glanzmann thrombasthenia
ITGB3	Glanzmann thrombasthenia
NBEAL2	Gray platelet syndrome
AP3B1	Hermansky-Pudlak syndrome
BLOC1S3	Hermansky-Pudlak syndrome
DTNBP1	Hermansky-Pudlak syndrome
HPS1	Hermansky-Pudlak syndrome
HPS3	Hermansky-Pudlak syndrome
HPS4	Hermansky-Pudlak syndrome
HPS5	Hermansky-Pudlak syndrome
HPS6	Hermansky-Pudlak syndrome
PLAUR	Urokinase plasminogen activator defects
MYH9	MYH9-related disorder
FLI1	Paris-Trousseau thrombocytopenia
ANO6	Scott syndrome
ANKRD26	Thrombocytopenia
RBM8A	Thrombocytopenia-absent radius syndrome
TBXA2R	Thromboxane A2 receptor defect
WAS	Wiskott-Aldrich syndrome
GATA1	X-linked thrombocytopenia with dyserythropoiesis
FGA	Disorders of fibrinogen
FGB	Disorders of fibrinogen
FGG	Disorders of fibrinogen

F2	Prothrombin deficiency
F5	Congenital factor V deficiency
F7	Congenital factor VII deficiency
F8	Hemophilia A
F9	Hemophilia B
F10	Congenital factor X deficiency
F11	Congenital factor XI deficiency
F13A1	Congenital factor XIII deficiency
F13B	Congenital factor XIII deficiency
SERPINE1	Congenital plasminogen activator inhibitor type 1 deficiency
VKORC1	Inherited deficiency of the vitamin K dependent clotting factors
VWF	Von Willebrand disease
SERPINA2	Alpha 2 antiplasmin deficiency
LMAN1	Combined V and VIII deficiency
MCFD2	Combined V and VIII deficiency
GGCX	Inherited deficiency of the vitamin K dependent clotting factors
