# Supplementary material for: Human phenotype ontology annotation and cluster analysis to unravel genetic defects in 707 cases with unexplained bleeding and platelet disorders
Source: Genome Med. 2015 Apr 9;7(1):36. doi: 10.1186/s13073-015-0151-5 (PMC4422517; doi:10.1186/s13073-015-0151-5)
Supplement: Additional file 8: — A table indicating recruitment to the BRIDGE-BPD study at the completion of Stage 1. [file 13073_2015_151_MOESM8_ESM.pdf]

Additional file 8: Stage 1 of the BRIDGE-BPD study.

	**Recruited and HPO annotated**	**Exome sequencing completed**
Index cases	648	471
Relatives	59	47
**Total**	**707**	**518**
