# Supplementary material for: Human phenotype ontology annotation and cluster analysis to unravel genetic defects in 707 cases with unexplained bleeding and platelet disorders
Source: Genome Med. 2015 Apr 9;7(1):36. doi: 10.1186/s13073-015-0151-5 (PMC4422517; doi:10.1186/s13073-015-0151-5)
Supplement: Additional file 9: — A figure showing the HPO terms used to describe abnormal platelet morphology. [file 13073_2015_151_MOESM9_ESM.pdf]

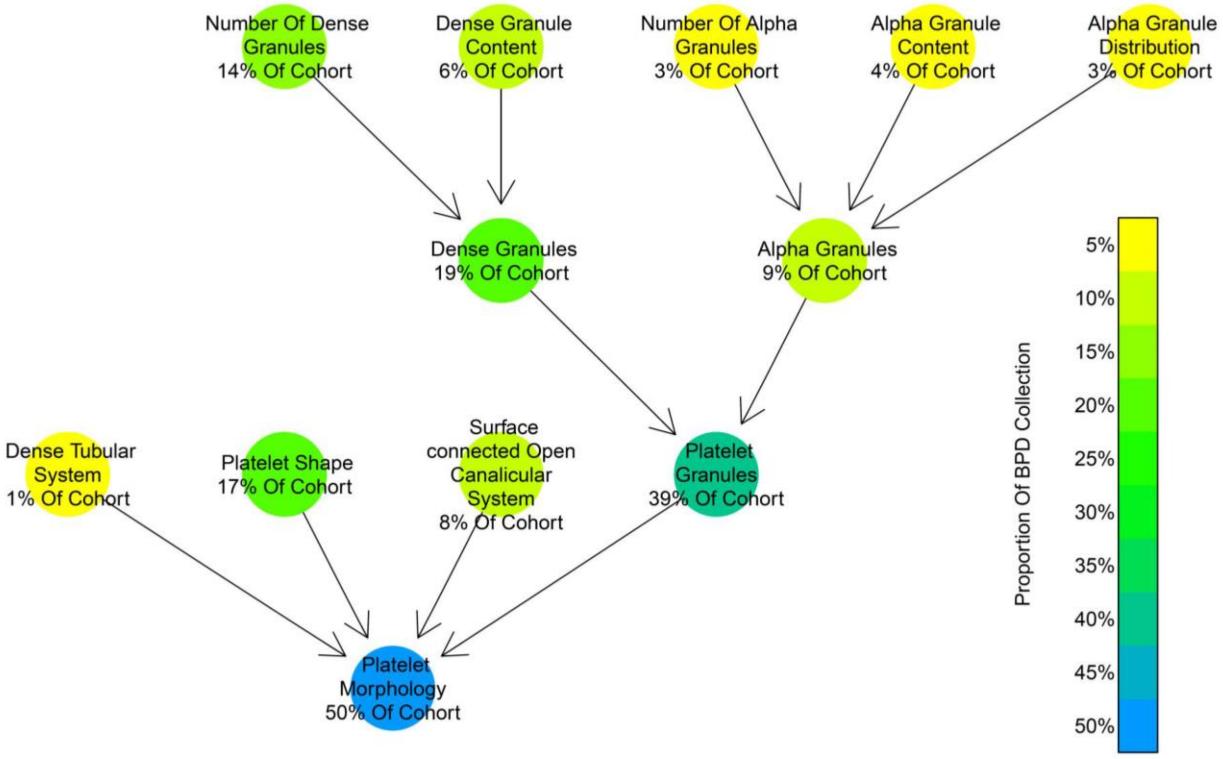

Additional file 9. HPO terms used to describe abnormalities of platelet morphology. Subgraph of the HPO showing the terms applied to the 50% of cases with abnormalities of platelet morphology determined by light or electron microscopy.
