# Supplementary material for: Human phenotype ontology annotation and cluster analysis to unravel genetic defects in 707 cases with unexplained bleeding and platelet disorders
Source: Genome Med. 2015 Apr 9;7(1):36. doi: 10.1186/s13073-015-0151-5 (PMC4422517; doi:10.1186/s13073-015-0151-5)
Supplement: Additional file 10: — A table listing 30 phenotype clusters identified in the BRIDGE-BPD cohort. [file 13073_2015_151_MOESM10_ESM.pdf]

Additional file 10. The principal HPO terms associated with each of the 30 clusters of cases obtained by PAM.

Cluster	Size	Terms
1	33	Increased mean platelet volume, thrombocytopenia
2	17	Intramuscular hematoma, myalgia, hypertension
3	21	Cholelithiasis, gastroesophageal reflux, gastrointestinal angiodysplasia
4	30	Allergic rhinitis, asthma, prenatal maternal abnormality
5	14	Abnormal bleeding
6	32	Hypertension, hematuria, hyperphosphatemia
7	25	Impaired arachidonic acid-induced platelet aggregation, impaired ADP-induced platelet aggregation, impaired collagen-induced platelet aggregation
8	6	Impaired ADP-induced platelet aggregation
9	35	Abnormality of coagulation
10	27	Depression, attention deficit hyperactivity disorder, velopharyngeal insufficiency
11	21	Abnormality of the hair, hypopigmentation of the skin, ocular albinism
12	23	Impaired thrombin-induced platelet aggregation, impaired collagen-induced platelet aggregation, impaired arachidonic acid-induced platelet aggregation
13	12	Thrombocytopenia
14	5	Abnormal platelet function
15	20	Growth delay, sensorineural hearing impairment, skeletal dysplasia
16	28	Increased serum lactate, increased susceptibility to fractures, joint hypermobility

17	36	Abnormality of erythrocytes, thrombocytopenia
18	15	Impaired epinephrine-induced platelet aggregation, impaired ADP-induced platelet aggregation
19	22	Abnormal thrombosis, lymphangioma, skeletal dysplasia
20	25	Giant platelets, thrombocytopenia, abnormal platelet shape
21	17	Abnormal platelet granules, impaired epinephrine-induced platelet aggregation, impaired ADP-induced platelet aggregation
22	15	Recurrent infections, asthma, abnormal platelet shape
23	27	Abnormality of leukocytes, thrombocytopenia, monocytopenia
24	36	Mental deterioration, epileptic spasms, hypertonia
25	20	Headache, abnormal dense granule content, immune dysregulation
26	20	Impaired thromboxane A2 agonist-induced platelet aggregation, abnormal number of dense granules, thrombocytosis
27	17	Impaired platelet adhesion, decreased platelet glycoprotein IIb-IIIa, decreased platelet glycoprotein Ib-IX-V
28	27	Abnormal alpha granule distribution, abnormal surface-connected open canalicular system, abnormal platelet shape
29	16	Autism spectrum disorder, thrombocytosis, decreased mean platelet volume
30	6	Abnormal platelet granules
