# Supplementary material for: Human phenotype ontology annotation and cluster analysis to unravel genetic defects in 707 cases with unexplained bleeding and platelet disorders
Source: Genome Med. 2015 Apr 9;7(1):36. doi: 10.1186/s13073-015-0151-5 (PMC4422517; doi:10.1186/s13073-015-0151-5)
Supplement: Additional file 11: — A table listing variants of uncertain significance identified in genes associated with autosomal recessive and X-linked recessive bleeding and platelet disorders. [file 13073_2015_151_MOESM11_ESM.pdf]

Additional file 11. Variants of uncertain significance identified in genes associated with autosomal recessive and X-linked recessive bleeding and platelet disorders.

Case	Position	Gene	Ref	Alt	Genotype	HGMD	Effect	Hematological HPO terms	Other HPO terms
B200020	1:169505902	*F5*	C	G	C|G	No	D1605H	Impaired collagen-induced platelet aggregation, impaired arachidonic acid-induced platelet aggregation, impaired ristocetin-induced platelet aggregation, impaired thromboxane A2 agonist-induced platelet aggregation, impaired ADP-induced platelet aggregation, impaired epinephrine-induced platelet aggregation, abnormal platelet granules, bleeding with minor or no trauma, subcutaneous hemorrhage, prolonged bleeding after dental extraction.	Gastric ulcer, uterine leiomyoma
	1:169511483	*F5*	A	C	A|C	No	L949V		
B200590	3:47045665	*NBEAL2*	G	A	G|A	No	D1994N	Hypofibrinogenemia, impaired ADP-induced platelet aggregation, impaired epinephrine-induced platelet aggregation, abnormal platelet granules, abnormal bleeding.	
	3:47049149	*NBEAL2*	C	A	C|A	No	P2490H		
B200017	3:128780950	*GP9*	C	T	T|T	No	P123L	Impaired ADP-induced platelet aggregation, impaired epinephrine-induced platelet aggregation, abnormal platelet granules, subcutaneous hemorrhage, epistaxis.	Recurrent abscess formation, asthma.
B200819	6:15663079	*DTNBP1*	G	A	A|A	No	R8W	Impaired ristocetin-induced platelet aggregation, increased mean platelet volume, thrombocytopenia, bleeding with minor or no trauma, subcutaneous hemorrhage, epistaxis, abnormal platelet	Abnormal facial shape, abnormality of the ear, abnormality of the palate, abnormality of the cardiovascular system,

								shape, abnormal alpha granule distribution, abnormal surface-connected open canalicular system.	intellectual disability.
B200027	12:6092332	*VWF*	T	A	T|A	No	R2355S	Reduced alpha/beta synthesis ratio, abnormal platelet granules, bleeding with minor or no trauma, subcutaneous hemorrhage, epistaxis, menorrhagia, prolonged bleeding after dental extraction.	Migraine.
	12:6204637	*VWF*	C	T	C|T	No	E216K		
B200065	12:6103193	*VWF*	G	A	G|A	Yes	P2145S	Subcutaneous hemorrhage, epistaxis, menorrhagia, post-partum hemorrhage, prolonged bleeding after dental extraction, abnormal platelet function.	Antiphospholipid antibody positivity, prenatal maternal abnormality, asthma, neoplasm of the breast.
	12:6125733	*VWF*	A	C	A|C	No	L1754V		
B200830	12:6125743	*VWF*	C	G	C|G	No	E1750D	Abnormal platelet function, bleeding with minor or no trauma, subcutaneous hemorrhage, epistaxis, menorrhagia, prolonged bleeding after surgery, prolonged bleeding after dental extraction, abnormal alpha granule distribution.	
	12:6128327	*VWF*	A	C	A|C	No	H1419Q		
	12:6172207	*VWF*	G	C	G|C	No	I482M		
B200391	X:154132714	*F8*	G	A	A	No	T1891I	Bone marrow hypocellularity, decreased platelet glycoprotein Ib-IX-V, thrombocytopenia, bleeding with minor or no trauma, subcutaneous hemorrhage, epistaxis, gastrointestinal hemorrhage, joint hemorrhage, prolonged bleeding after surgery, prolonged bleeding after dental extraction, abnormal platelet shape, abnormal number of alpha granules.	Splenomegaly, intramuscular hematoma.
B200443	X:154157563	*F8*	T	G	G	No	K1501T	Impaired ADP-induced platelet aggregation, bleeding with minor or no	

								trauma, spontaneous hematomas, abnormal number of dense granules, reduced factor IX activity.	
B200616	X:154159729	*F8*	G	C	C		No	T779R	Thrombocytopenia, bleeding requiring red cell transfusion, prolonged bleeding after dental extraction, abnormal platelet granules, subcutaneous haemorrhage.	Diabetes mellitus.

Abbreviations: Ref, reference; Alt, alternative. *Effect considered relative to the Consensus Coding Sequence (CCDS) for each gene. In each case, the phenotype was classified as *not explained*.
