# Supplementary material for: Human phenotype ontology annotation and cluster analysis to unravel genetic defects in 707 cases with unexplained bleeding and platelet disorders
Source: Genome Med. 2015 Apr 9;7(1):36. doi: 10.1186/s13073-015-0151-5 (PMC4422517; doi:10.1186/s13073-015-0151-5)
Supplement: Additional file 12: — A table listing additional members of the BRIDGE-BPD Consortium. [file 13073_2015_151_MOESM12_ESM.pdf]

Additional file 12. Additional members of the BRIDGE-BPD Consortium.

Name	Affiliation
Cornelius Albers	Department of Haematology, University of Cambridge, Cambridge, UK.
Paula Bolton-Maggs	NHS Blood and Transplant, Manchester, UK.
Martijn Breuning	Department of Clinical Genetics, Leiden University Medical Centre, Leiden, the Netherlands.
James Bussel	Pediatric Haematology-Oncology, New York-Presbyterian/Weill Cornell, New York, USA.
Peter Collins	Cardiff Institute of Infection and Immunity, University of Cardiff, Cardiff, UK.
Cedric Ghevaert	Department of Haematology, University of Cambridge, Cambridge, UK.
Andreas Greinacher	Institut für Immunologie und Transfusionsmedizin Universitätsmedizin Ernst-Moritz-Arndt Universität, Greifswald, Germany
Johan Heemskerk	Thrombosis and Haemostasis, Maastricht University, Maastricht, the Netherlands.
Yvonne Henskens	Department of Haematology, Maastricht University Medical Centre, Maastricht, the Netherlands.
Myrto Kostadima	Department of Haematology, University of Cambridge, Cambridge, UK.
Mary Mathias	Department of Haematology, Great Ormond Street Hospital for Children NHS Trust, London, UK.
Ruth Newbury Ecob	Department of Clinical Genetics, St Michael’s Hospital, Bristol, UK.
Matthew Rondina	George and Dolores Eccles Institute of Human Genetics, University Health Care - Internal Medicine, Salt Lake City, USA.
Maria Vries	Department of Haematology, Maastricht University Medical Centre, Maastricht, the Netherlands.
